# Stent Deployment Without Tract Dilation in Endoscopic Ultrasound-Guided Hepaticogastrostomy Using a Novel Partially Covered Metal Stent With a Super-Slim Stent Delivery System: A Case Report

**DOI:** 10.7759/cureus.60406

**Published:** 2024-05-16

**Authors:** Koji Takahashi, Hiroshi Ohyama, Izumi Ohno, Naoya Kato

**Affiliations:** 1 Department of Gastroenterology, Chiba University, Chiba, JPN; 2 Department of Medical Oncology, Chiba University, Chiba, JPN

**Keywords:** novel, delivery system, partially covered metal stent, endoscopic ultrasound-guided hepaticogastrostomy, tract dilation

## Abstract

Endoscopic ultrasound-guided hepaticogastrostomy is performed when transpapillary biliary drainage using endoscopic retrograde cholangiopancreatography is difficult due to surgically altered anatomy, an inaccessible papilla, or difficult biliary cannulation. This procedure consists of puncturing the intrahepatic bile duct from the stomach, inserting a guidewire into the bile duct, dilating the puncture tract, and placing a stent. Recently, a novel partially covered self-expandable metal stent with a super-slim stent delivery system of 5.9 Fr has become available. With this stent, endoscopic ultrasound-guided hepaticogastrostomy can be performed without using a dilator to expand the puncture tract. Herein, we describe a technique for dilator-free stent deployment for endoscopic ultrasound-guided hepaticogastrostomy using this novel stent. We performed an endoscopic ultrasound-guided hepaticogastrostomy with this stent in a 65-year-old patient with obstructive jaundice due to pancreatic head cancer without adverse events and with satisfactory improvement in jaundice. This procedure is expected to reduce bile leakage into the abdominal cavity and shorten the procedure time.

## Introduction

Transpapillary biliary drainage using endoscopic retrograde cholangiopancreatography (ERCP) is the first choice for biliary drainage, but if this is difficult for some reason, endoscopic ultrasound-guided hepaticogastrostomy (EUS-HGS) is performed. Such reasons include surgically altered anatomy, an inaccessible papilla, and difficult biliary cannulation [1,2]. EUS-HGS is a method for performing biliary drainage by placing a stent from the stomach into the intrahepatic bile duct using an ultrasound-guided endoscope. It is useful when transpapillary biliary drainage is difficult, but bile leakage into the peritoneal cavity may occur, and it can lead to adverse events such as biliary peritonitis [3-5]. Possible measures to reduce bile leakage into the abdominal cavity during the procedure include performing the procedure quickly and reducing the dilated diameter of the puncture tract. Recently, a novel partially covered self-expandable metal stent with a super-slim delivery system (HANAROSTENT® Biliary Partial Cover Benefit™; M.I. Tech, Seoul, South Korea) has become available. This stent delivery system is 5.9 Fr, and the tip is extremely tapered. With this stent, EUS-HGS can be performed without using a dilator to expand the puncture tract. Herein, we describe a dilator-free stent deployment technique for EUS-HGS using this novel stent.

## Case presentation

**Figure 1:**
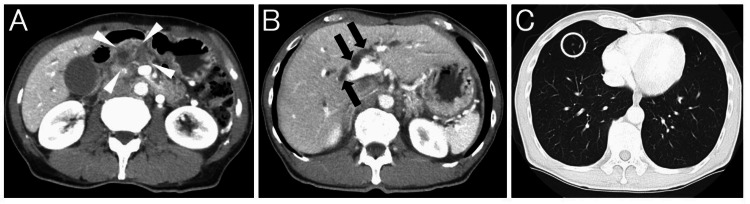
Contrast-enhanced computed tomography scan images at the time of the visit to our hospital. A: A tumor is found in the pancreatic head (white arrowhead). B: Bile duct upstream of the tumor is dilated (black arrow). C: A tumor is also found in the lung (circled).

A 65-year-old man noticed that his urine had become darker and visited his family hospital. Blood tests showed elevated hepatobiliary enzymes and jaundice. A non-contrasted computed tomography (CT) scan revealed a dilated bile duct and he was referred to our hospital. The patient has had type 2 diabetes for 30 years and was diagnosed with chronic pancreatitis 20 years ago. There is no other notable medical history for this patient. At the time of our visit, the patient had mild abdominal pain and jaundice. Contrast-enhanced CT scan revealed a tumor in the pancreatic head and resulting distal bile duct obstruction. Tumors were also found in the lungs (Figure 1).

**Figure 2:**
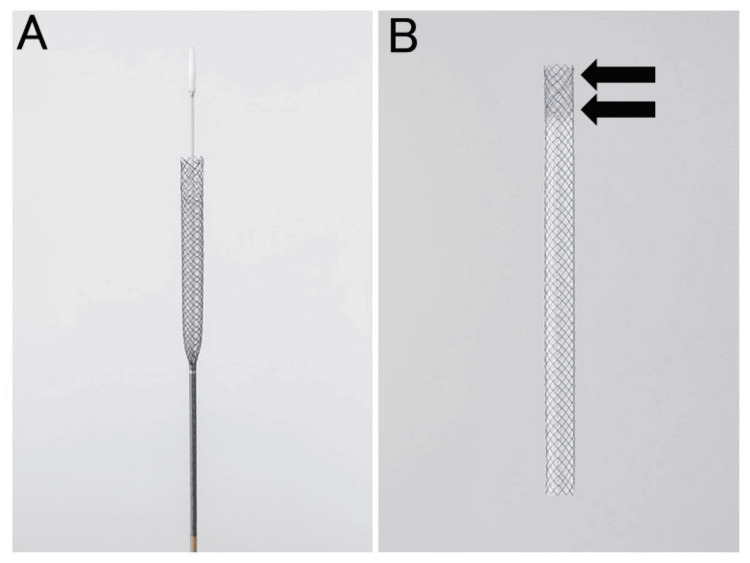
A novel partially covered self-expandable metal stent with a fine-gauge stent delivery system (HANAROSTENT® Biliary Partial Cover Benefit™; M.I. Tech, Seoul, South Korea). A: The stent is mounted on a 5.9 Fr delivery system. B: The stent is uncovered at the tip (arrow) and the rest is covered.

**Figure 3:**
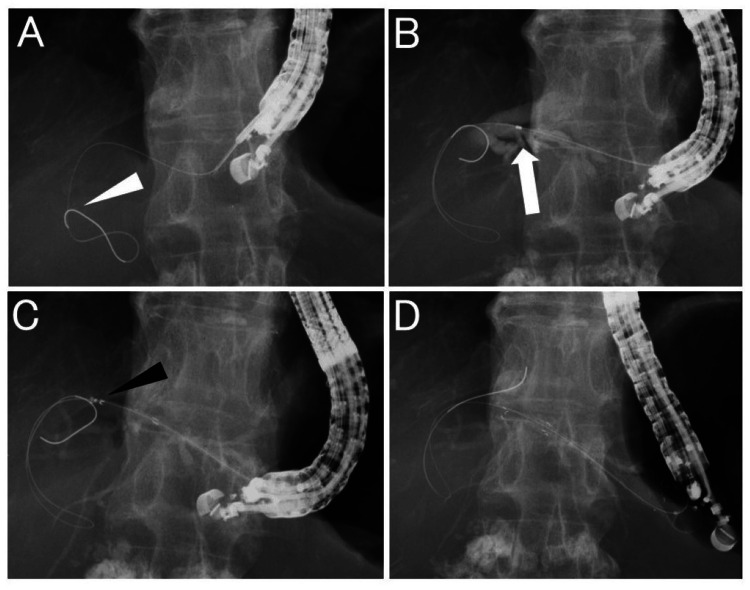
Fluoroscopic image during stent placement. A: The intrahepatic bile duct is punctured using a 19-gauge needle, and the guidewire (white arrowhead) is inserted into the bile duct. B: The contrast catheter for endoscopic retrograde cholangiopancreatography (white arrow) is inserted into the bile duct and the contrast medium is injected. C: The stent delivery (black arrowhead) is inserted into the bile duct without puncture tract dilation. D: Stent deployment and placement is successfully finished.

He was admitted for tissue sampling of the pancreatic head tumor and biliary drainage. Endoscopic ultrasound-guided fine needle aspiration (EUS-FNA) was performed the day after admission. Subsequently, we attempted biliary drainage by ERCP. We attempted bile duct intubation through the duodenal papilla using an ERCP-catheter but were unable to insert the catheter into the bile duct. We extracted the scope for ERCP and inserted an EUS scope, intending to switch to EUS-HGS. The left lobe of the liver was visualized from within the stomach with echo imaging, the lateral segmental branch of the intrahepatic bile duct was punctured with a 19-gauge needle (EZ Shot3 Plus; Olympus Corporation, Tokyo, Japan), and a 0.025-inch guidewire (EndoSelector; Boston Scientific Corporation, Marlborough, MA, USA) was inserted into the intrahepatic bile duct. Next, an ERCP-catheter (MTW ERCP-catheter; MTW Endoskopie Manufaktur, Wesel, Germany) with 2.3 mm diameter was inserted into the intrahepatic bile duct, and after bile aspiration and cholangiography, a stent delivery system (HANAROSTENT® Biliary Partial Cover Benefit™, 8mm × 12cm; Olympus) (Figure 2) was inserted, deployed, and placed without tract dilation (Figure 3).

**Figure 4:**
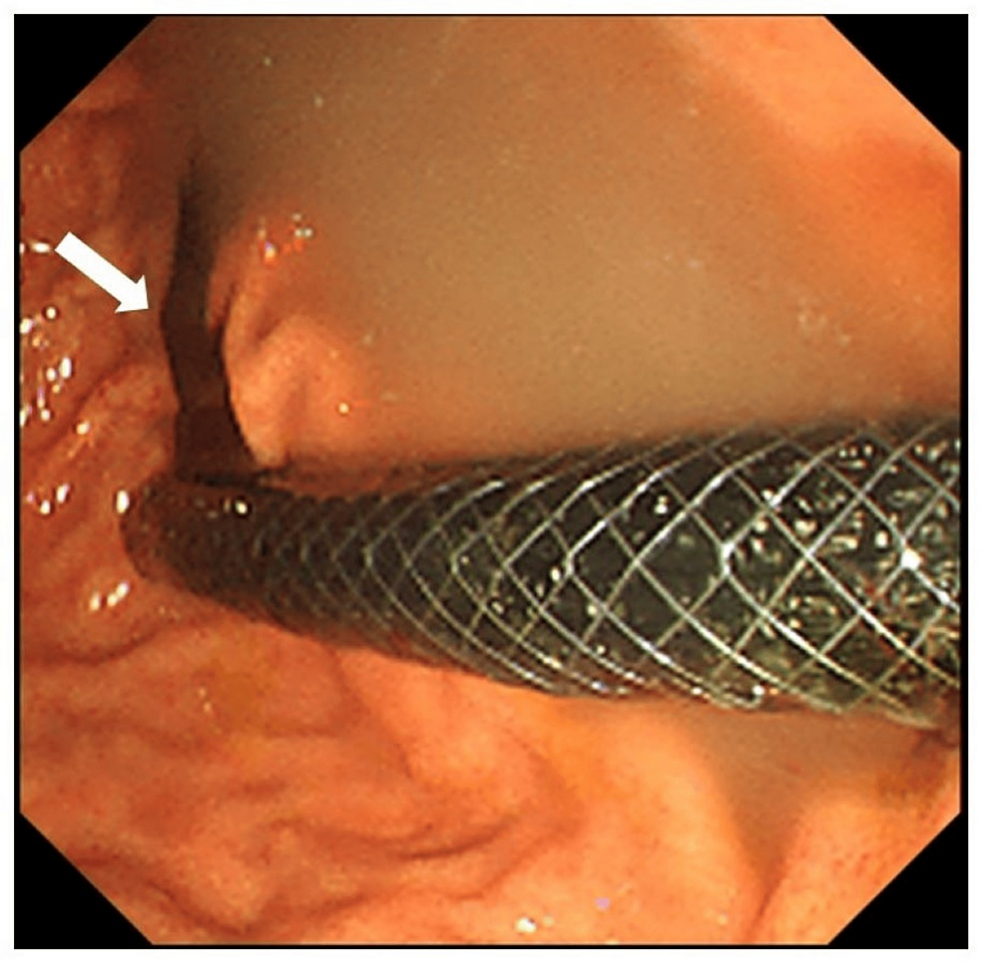
Endoscopic image of the stomach after stenting. Bile (arrow) is drained from the stent into the stomach after stent placement.

After stent placement, bile was allowed to drain into the stomach (Figure 4).

**Figure 5:**
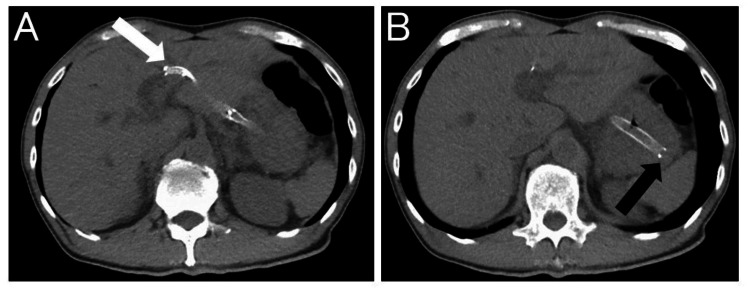
A CT scan on the next day showed no displacement of the placed stent. A: The tip of the stent (uncovered side) is located within the intrahepatic bile duct (white arrow). B: The posterior end of the stent is located within the stomach (black arrow).

A CT scan the next day showed no displacement of the placed stent (Figure 5).

**Figure 6:**
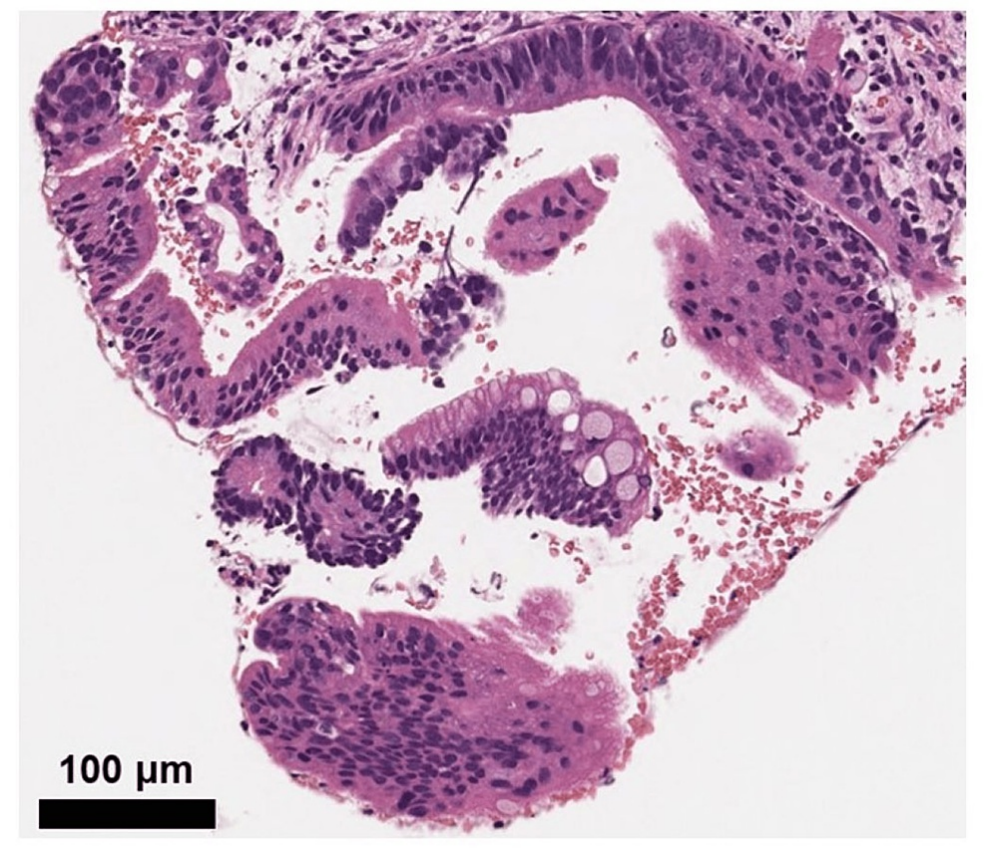
The histopathologic diagnosis in the endoscopic ultrasound-guided fine needle aspiration specimen was adenocarcinoma.

The patient had no adverse events and was discharged from the hospital 5 days after treatment. The histopathologic diagnosis in the EUS-FNA specimen was adenocarcinoma (Figure 6).

He was diagnosed with pancreatic head cancer with lung metastasis. After confirming that jaundice had disappeared, systemic chemotherapy was started 19 days after EUS-HGS. It has been about a month since the stent was implanted, but the patient has been progressing without any stent-related problems and continues to receive systemic chemotherapy.

## Discussion

This novel stent is a metal stent with 15 mm of the stent tip uncovered and the rest covered, available in 6 mm and 8 mm diameters, and a 5.9 Fr delivery system. Two types of metal stents are used in EUS-HGS: fully covered and partially covered. Fully covered type stents are removable after placement but may cause displacement, while partially covered stents are less prone to displacement, but are difficult to remove. The delivery systems of covered metal stents are often 8 Fr or more and require puncture tract dilatation prior to stenting, but this new stent was mounted in a delivery system with a narrower diameter of 5.9 Fr and extremely tapered tips. Therefore, it can be placed without dilation of the puncture tract.

In general, EUS-HGS consists of puncturing the intrahepatic bile duct from the stomach, contrasting the bile duct and inserting a guidewire, dilating the puncture tract, inserting an ERCP-catheter into the bile duct, aspirating bile, and placing a stent. In EUS-HGS, aspirating bile during the procedure results in less bile leakage [6]. Plastic and metal stents are available for implantation. Plastic stents generally have a diameter of 7 Fr or 8.5 Fr; therefore, puncture tract dilatation is almost always required for plastic stent implantation. However, there is a report that a newer, tapered, 7 Fr plastic stent did not require tract dilation [7].

Recently, metal stents with small-diameter delivery systems have appeared. There have been reports of metal stenting in EUS-HGS without puncture tract dilation using these stents [8-10]. When the puncture tract is dilated, bile is more likely to leak from the puncture tract into the abdominal cavity. In addition, when performing puncture tract dilatation, the action of pushing the dilator may create a distance between the stomach and the liver, making subsequent stent insertion difficult. Another advantage of not performing the tract dilation is that it reduces the number of instrument insertions during the procedure, leading to shorter procedure times.

In our case, no adverse events occurred after the treatment, the jaundice showed signs of improvement, and systemic chemotherapy could be started. EUS-HGS may cause bile leakage into the abdominal cavity, whereas this stent can be placed quickly without the need to dilate the puncture tract. Because this is a partially covered metal stent, misalignment is unlikely. EUS-HGS with this stent, which does not require puncture tract dilation, is a very useful method.

## Conclusions

We have described a technique for EUS-HGS without puncture dilation using a partially covered metal stent in a novel, super-slim delivery system. EUS-HGS with this novel stent does not require puncture tract dilatation, thus reducing the possibility of bile leaking into the abdominal cavity. In addition, the lack of a dilator leads to a shorter procedure time compared with the conventional method. Although the stent tip needs to be well positioned, this stent could make the EUS-HGS procedure safer and simpler.
